# The Overcrowded Crossroads: Mitochondria, Alpha-Synuclein, and the Endo-Lysosomal System Interaction in Parkinson’s Disease

**DOI:** 10.3390/ijms20215312

**Published:** 2019-10-25

**Authors:** Kai-Jung Lin, Kai-Lieh Lin, Shang-Der Chen, Chia-Wei Liou, Yao-Chung Chuang, Hung-Yu Lin, Tsu-Kung Lin

**Affiliations:** 1 Mitochondrial Research Unit, Kaohsiung Chang Gung Memorial Hospital and Chang Gung University College of Medicine, Kaohsiung 833, Taiwan; b101101092@tmu.edu.tw (K.-J.L.); 100311039@gms.tcu.edu.tw (K.-L.L.); jp1916@ms4.hinet.net (S.-D.C.); cwliou@ms22.hinet.net (C.-W.L.); ycchuang@adm.cgmh.org.tw (Y.-C.C.); linhungyu700218@gmail.com (H.-Y.L.); 2 Department of Anesthesiology, Kaohsiung Chang Gung Memorial Hospital and Chang Gung University College of Medicine, Kaohsiung 833, Taiwan; 3 Department of Neurology, Kaohsiung Chang Gung Memorial Hospital and Chang Gung University College of Medicine, Kaohsiung 833, Taiwan; 4 Center of Parkinson’s Disease, Kaohsiung Chang Gung Memorial Hospital and Chang Gung University College of Medicine, Kaohsiung 833, Taiwan

**Keywords:** Parkinson’s disease, Lewy body, mitochondria, mitophagy, α-synuclein, lysosome

## Abstract

Parkinson’s disease (PD) is the second most common neurodegenerative disorder worldwide, mainly affecting the elderly. The disease progresses gradually, with core motor presentations and a multitude of non-motor manifestations. There are two neuropathological hallmarks of PD, the dopaminergic neuronal loss and the alpha-synuclein-containing Lewy body inclusions in the substantia nigra. While the exact pathomechanisms of PD remain unclear, genetic investigations have revealed evidence of the involvement of mitochondrial function, alpha-synuclein (α-syn) aggregation, and the endo-lysosomal system, in disease pathogenesis. Due to the high energy demand of dopaminergic neurons, mitochondria are of special importance acting as the cellular powerhouse. Mitochondrial dynamic fusion and fission, and autophagy quality control keep the mitochondrial network in a healthy state. Should defects of the organelle occur, a variety of reactions would ensue at the cellular level, including disrupted mitochondrial respiratory network and perturbed calcium homeostasis, possibly resulting in cellular death. Meanwhile, α-syn is a presynaptic protein that helps regulate synaptic vesicle transportation and endocytosis. Its misfolding into oligomeric sheets and fibrillation is toxic to the mitochondria and neurons. Increased cellular oxidative stress leads to α-syn accumulation, causing mitochondrial dysfunction. The proteasome and endo-lysosomal systems function to regulate damage and unwanted waste management within the cell while facilitating the quality control of mitochondria and α-syn. This review will analyze the biological functions and interactions between mitochondria, α-syn, and the endo-lysosomal system in the pathogenesis of PD.

## 1. Introduction

Parkinson’s disease (PD) is the second most common neurodegenerative disease in the world, affecting up to two percent of the population over 65 years of age [1]. Age has been recognized as the greatest risk factor affecting disease progression, with evidence of a doubling of the risks of mortality and dementia post PD diagnosis [2]. With an aging global population, the significance of this disease is accentuated. James Parkinson first described clinical manifestations of bradykinesia, rigidity, rest tremor, and gait disturbance over two centuries ago [3]. A century later, the pathological hallmark of α-synuclein (α-syn) protein aggregates, termed Lewy bodies, was discovered in the substantia nigra pars compacta (SNpc) by Tre´tiakoff [4]. By the 1960s, high-dose L-DOPA was introduced as the standard clinical treatment which greatly improved the quality of life and the life expectancy of PD patients [5]. Recent clinical-pathological studies have provided insight into another important PD hallmark, progressive neuronal loss. Based on the PD progression staging system proposed by Braak et al., neurodegeneration begins and is initially restricted to the ventrolateral region of the SNpc, becoming more widespread by the end-stage [6]. Studies have also demonstrated reciprocal Lewy body accumulation traveling from both the peripheral and enteric nervous systems to the central nervous system [7,8]. This corresponds with heterogeneous non-motor PD features which have been shown to occur up to decades in advance of motor symptoms. The non-motor manifestations include altered endocrine signaling, autonomic control, neuropsychiatric functions, olfactory functions, gastrointestinal motility, sleep disturbance, and pain perception [9,10]. While the pathogenesis of PD has been investigated extensively, the primary causes remain elusive; although many risk factors have been identified, including age, environmental toxin exposure, and genomic defects [11,12]. More recent investigations into discrete genetic and molecular causes have implicated mitochondrial dysfunction, oxidative stress, calcium (Ca^2+^) homeostasis, autophagy and endo-lysosomal system dysfunction, the accumulation of aberrant or misfolded proteins, and ubiquitin-proteasome system dysfunction in principle molecular pathways as underlying the pathogenesis of sporadic and familial PD [13,14,15]. Furthermore, reports indicate a trilateral relationship of mitochondrial dysfunction, α-syn aggregation, and degradation of the endo-lysosomal and proteasome systems in the neurodegenerative process of PD [16,17,18]. 

**Figure 1:**
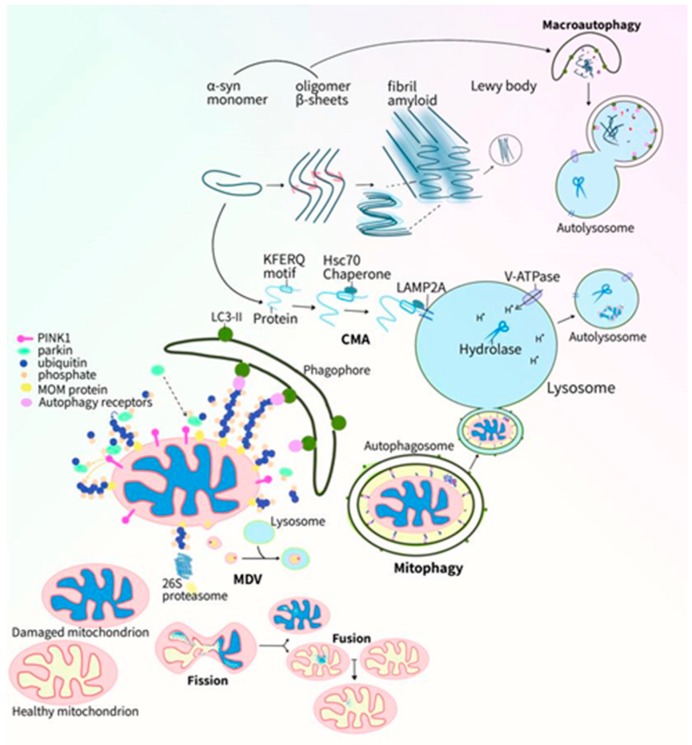
The importance of mitochondrial dysfunction, α-syn aggregation, and the autophagy-endo-lysosomal system dysregulation in PD (Parkinson’s disease) pathogenesis. The clearance of damaged mitochondria and denatured α-synuclein (α-syn) are through autophagy-lysosome pathways. Minor mitochondrial damage is fixed via dynamic fission and fusion, complementing the damaged organelles and mitochondrial proteins. Medium amounts of damaged mitochondrial proteins and mitochondria parts are delivered to the lysosome via mitochondrial-derived vesicles (MDVs). The whole mitochondrion is trafficked to the lysosome for degradation via the mitophagy process (this figure depicts the most well-known PINK1/parkin dependent mitophagy pathway). Mitochondrial membrane potential dissipation leads to PINK1 kinase stabilization on the mitochondrial outer membrane (OM) and recruits cytosolic E3 ubiquitin ligase, parkin, to the mitochondria. Parkin subsequently ubiquitinates mitochondrial OM proteins, tagging them for autophagy receptors (such as p62) recognition. These autophagy receptors bind with LC3-II-positive phagophores and the double-membraned structure closes up around the mitochondrion to form autophagosomes. Autophagosomes eventually fuse with lysosomes to form autolysosomes where damaged mitochondrion is degraded. Upon initiation of the PINK1/parkin dependent pathway, the dynamic fusion and motility of the damaged mitochondrion is disabled by targeting Mitofusin (Mfn) and Miro for ubiquitin-proteasomal degradation. Native α-syn monomers are able to transition into toxic beta-sheet containing oligomers, which further converts into insoluble amyloid fibrils and are eventually deposited into Lewy bodies. α-syn monomers are degraded via the chaperone mediated autophagy (CMA) under physiological conditions. In this process, the heat shock cognate 71 kDa protein (Hsc70) chaperone recognizes the KFERQ domain of α-syn and targets the protein for the lysosome. At the lysosome membrane, the lysosome-associated membrane protein type 2A (LAMP2A) receptor assists in α-syn docking and internalization into the lysosome, where α-syn is degraded by hydrolases. Toxic α-syn oligomers and non-toxic monomers can both be degraded via the macroautophagy process.

In a normal-functioning neuron, mitochondria supply cellular adenosine triphosphate (ATP) via the respiratory chain, maintain cellular Ca^2+^ homeostasis, and regulate cellular growth and death [19]. The integrity of this organelle is maintained by its dynamic functions, including morphological changes of fusion and fission, and quality control mechanisms [20]. The constant production of ATP through the I–IV respiratory chain enzymatic complexes located in the mitochondrial inner membrane is coupled with leaked electrons leading to the generation of toxic reactive oxygen species (ROS). Protective antioxidative manganese superoxide dismutase (SOD2) enzymes reside in the mitochondria and work to dismutate the radical superoxide anions. Oxidative stress occurs when imbalances between free radical production and the antioxidative system develop, leading to macromolecular damage and further cellular functional impairment [21]. Meanwhile, the PD-related denaturing of the pathological protein α-syn also occurs. This 140-amino-acid protein is normally soluble and functions as a presynaptic terminal protein assisting with synaptic vesicle trafficking and endocytosis. Of particular importance to dopaminergic (DA) neurons, α-syn regulates the synthesis, storage, and release of dopamine [22]. Furthermore, α-syn interacts with membrane lipids of mitochondria and the endoplasmic reticulum (ER), affecting Ca^2+^ homeostasis. Through interactions with tubulin, α-syn contributes to microtubule dynamics [17,23]. A normal turnover of mitochondria and α-syn contribute to neuronal health, while mechanisms of their homeostasis involve the proteasome pathways and autophagy and endo-lysosomal pathways. Misfolded proteins are targeted for protease degradation, and larger sized waste is segregated and degraded by lysosomal hydrolases. When imbalances occur, for instance by mitochondrial defects, mitochondrial ROS production increases which promote intracellular α-syn misfolding and aggregation [24,25]. Studies have shown α-syn aggregation to cause a multitude of mitochondrial defects, including decreased mitochondrial membrane potential and energy production, disruption of mitochondrial-ER Ca^2+^ homeostasis, inhibited mitochondrial dynamics, and induced mitochondrial pro-apoptotic protein cytochrome c release [26,27]. Increased cellular ROS triggers mitochondrial importation of α-syn, subsequently inducing intra-mitochondrial α-syn aggregation and respiratory Complex I dysfunction. Overexpression or mutation of α-syn will impair mitochondrial functions, while subsequent increased ROS will drive toxic α-syn conformation, thereby creating a vicious cycle [18,28]. The build-up of damaged mitochondria and α-syn increases the proteasome and autophagy-lysosomal loads, possibly causing dysfunctions in these clearance pathways [29,30]. On the contrary, when degradation pathways are disrupted, possibly due to genetic mutations, the normal quality control process is hindered resulting in a build-up of unwanted proteins and fragmented mitochondria. This perturbed degradation impairs the biogenesis of mitochondria and mitochondrial proteins and induces mitochondrial stress responses such as the mitochondrial unfolded protein response (UPR^mt^) and apoptotic mechanisms [31,32]. The decreased degradation of α-syn may lead to α-syn misfolding, oligomerization, and fibrillization. These toxic structures cause further mitochondrial dysfunction which again may lead to cellular death. There has also been evidence of prion-like propagation of α-syn between neuronal synapses corresponding with the progressive symptoms of PD [33,34]. The associations between PD and endo-lysosomal dysfunction have been observed in mitochondrial quality control, intracellular protein regulation, intra- and inter-cellular α-syn transfer, genetic crosstalk between PD and lysosomal dysfunctions, and clinical lysosomal disease presentations [35]. Of note, clinical associations have been observed in Gaucher’s disease (GD) patients with a presentation of parkinsonism and pathological Lewy body accumulations [36]. Following studies of this lysosomal storage disorder revealed that the GD causative gene *GBA1* also increased the risk for developing PD [37]. DA neurons produce and consume a great amount of ATP within the human body due to the millions of synapse connections they form, further emphasizing the importance of mitochondria in neuronal health [38,39,40]. The genetic defects of this organelle combined with the α-syn encoding gene and lysosomal genetic mutations are major causes of PD-related neurodegeneration [41]. We herein summarize the complex and interrelated roles of mitochondrial dysfunction, α-syn, and the endo-lysosomal system in PD neurodegeneration (Figure 1).

## 2. Mitochondria and Parkinson’s Disease

The earliest report of indicating an association between mitochondria and PD was in 1976, when scientists discovered a link between recreational drug use and contaminated mitochondrial Complex I inhibitor 1-methyl-4-phenyl-1,2,3,6-tetrahydropyridine (MPTP), resulting in PD-like motor symptoms. The additional observation of a 30% to 40% decrease in the mitochondrial Complex I activity observed in the SNpc of patients with PD further supports the critical role of this organelle in the development of PD [42]. The discovery that exposure to environmental mitochondrial electron transport chain (ETC) inhibitors including pesticides were parkinsonism-related further associated mitochondria with this disease. Subsequent genomic studies have also revealed many PD-related genetic mutations cause organelle dysfunction through disrupting the mitochondrial respiratory chain complexes, decreasing the mitochondrial membrane potential, causing mitochondrial fission, fusion, and quality control disequilibrium, affecting the autophagic degradation of the organelles’ damaged components, and activating the mitochondrial-related cell death signals through apoptosis. Since mitochondria are the major source of ROS under normal physiological conditions, the relevance of mitochondrial health for maintenance of organelle function is critical for DA neurons which are sensitive to oxidative damage, under additional ROS load from dopamine metabolism, and possess a relative paucity of antioxidative enzymes [43]. In the following sections, we will further discuss mitochondrial biology, homeostasis of intracellular Ca^2+^, apoptosis, the maintenance of mitochondrial quality, and recent hereditary PD genetic findings related to mitochondria. 

### 2.1. Mitochondrial Biology

Mitochondria are double-membraned, highly dynamic organelles that are responsible for efficient cellular energy generation, and mitochondrial functionality determines cellular survival or death [44]. The mitochondrial outer membrane (OM) and the mitochondrial inner membrane (IM) partition the mitochondria into two fluid-filled compartments. The outer compartment is the intermembrane space (IMS). The central compartment is the mitochondrial matrix, which is surrounded by convoluted IM folds known as cristae. This compartmentalized structure of the organelle enables bioenergetic catabolic processes and biosynthesis of nucleotides, glucose, amino acids, fatty acids, cholesterol, and heme. An estimated 90% of mammalian oxygen consumption is utilized by this organelle to produce adenosine triphosphate (ATP) via the chemiosmotic-coupling oxidative phosphorylation (OXPHOS) process [45]. Reduced equivalents (NADH and FADH_2_) generated from the tricarboxylic acid (TCA) cycle pass electrons on to the electron transport chain (ETC) located in the IM. The ETC enzyme Complexes I–IV use energy generated from the redox reactions to pump protons across the IM into the IMS. Impermeable to protons, the IM acts as a functional barrier and establishes an electrochemical gradient across the IM, termed mitochondrial membrane potential (Δψm) [46]. This proton gradient generated from electron transportation (oxidation) then drives the proton across the IM through the F1F0-ATP synthase (Complex V) and ultimately phosphorylate adenosine diphosphate (ADP) into ATP (phosphorylation) [47]. The mitochondrial proteome encompasses more than 1000 proteins and more than 95% are encoded by nuclear genes, synthesized on cytosolic ribosomes, while less than 5% are encoded by mitochondrial DNA (mtDNA) which are critical parts of the OXPHOS complexes. Each human cell harbors from a few hundred to one thousand mitochondria, and each mitochondrion contains two to eight copies of mtDNA in the matrix [48]. The 16569 bp double-stranded circular mtDNA contains 37 genes and encodes for 22 transfer RNAs (tRNA), the 12S and 16S ribosomal RNAs (rRNA), and 13 polypeptides that form core parts of the OXPHOS complexes [49]. 

There is a constant “leak” of electrons from the ETC, especially from Complexes I and III, to oxygen which forms superoxide anions radical (O_2_^•-^). The dismutation of O_2_^•-^ produces hydrogen peroxide (H_2_O_2_) and diffuses into the cytosol and nucleus to be detoxified by glutathione peroxidase and catalase into water [50]. In the presence of transition metals, H_2_O_2_ can then be turned into highly reactive hydroxyl radical (•OH) [51]. To cope with the ROS generated during metabolic processes, cells have developed the antioxidative system, including antioxidants and antioxidative enzymes. Physiological ROS levels are essential for cellular survival and act as important signals in a wide array of cellular responses by changing the redox state of signaling proteins regulating processes including altering mitochondrial morphology, quality control mitophagy, and mitochondrial bioenergetics [24,52,53]. However, when the optimum balance between ROS production and antioxidative capability is not achieved, the cellular redox states can be disturbed, leading to oxidative stress [54]. An excess of these chemically-active ROS can readily oxidize proteins, lipids, carbohydrates, DNA and RNA, and causes oxidative damage [51]. Since mitochondria have a less efficient DNA repair system, chronic exposure to ROS contributes to a more rapid accumulation of mtDNA mutations compared to nuclear DNA (nDNA), which has been linked to PD [13]. As mitochondria age, damage to proteins including α-syn and mtDNA perturb their normal function and energy states which may further lead to cell death and has been implicated in several age-related disorders [55,56].

### 2.2. Mitochondria, Intracellular Calcium Homeostasis, Apoptosis and Mitochondrial Dynamics

Another important function of mitochondria is in the homeostasis of intracellular Ca^2+^ and programmed cell death, both of which are highly relevant to PD [57,58,59]. Mitochondrial Ca^2+^ coordinates the upregulation of the OXPHOS machinery, while the dysregulated increase of Ca^2+^ could disrupt ATP synthesis and increase ROS production [60,61]. The close proximity of mitochondria with Ca^2+^ channels in the ER at the mitochondria-associated membranes allows transient influx from the intracellular Ca^2+^ stores to the mitochondrial matrix and assist in prompt mitochondrial Ca^2+^ buffering and Ca^2+^ gradient control in other cellular compartments [62,63,64,65]. Local Ca^2+^ sensors in the mitochondria IMS control Ca^2+^ uptake and signal mitochondrial trafficking along intracellular microtubules. Upon sensor signaling, Ca^2+^ influx from the cytosol passes through the voltage-dependent anion channel (VDAC) in the OM, the Δψm-driven mitochondrial Ca^2+^ uniporter (MCU) complex in the IM and finally enters the mitochondrial matrix [66]. Efflux of Ca^2+^ from the matrix is managed by the IM Na^+^/Ca^2+^ antiporter and normal brief flickering of IM mitochondrial permeability transition pore (mPTP) between open and closed states [67]. mPTP is also associated with ROS release to cytosol from the mitochondrial matrix, possessing timely and adaptive housekeeping functions by relieving mitochondria of potentially toxic accumulations of Ca^2+^ and ROS [68]. Upon extreme overloading of matrix Ca^2+^ and ROS in combination with high phosphate concentrations, there is an activation of cyclophilin D which induces sustained mPTP opening and leads to Δψm collapse, mitochondrial swelling, pro-apoptotic mediators release from the mitochondria matrix into the cytosol, and subsequent cell death via the intrinsic mitochondrial apoptotic pathway [51]. The implications of mitochondrial involvement in the intrinsic apoptotic pathway are associated with PD, while neuronal loss is a pathological hallmark of PD [69]. The intrinsic apoptosis pathway is initiated when the intracellular environment becomes too hazardous for cell survival and begins with the activation of the mitochondrial outer membrane permeabilization (MOMP) [70]. MOMP entails the opening of mPTP, with the release of mitochondrial pro-apoptotic proteins into the cytosol including cytochrome c. Activation of downstream apoptotic peptidase-activating factor 1 (APAF1), pro-caspase-9 (CASP9), and apoptosome-dependent activation of CASP 3 execute the cleavage and destruction of subcellular structures [71,72]. Mitochondria involvement is integrated within the apoptotic signal transduction cascades and ensure the process of a controlled type of cell death, with minimal effect on surrounding tissue [73]. Of note, the overactivation of caspases can lead to excessive cell death, which is observed in PD [74]. Thus, maintenance of a healthy pool of mitochondria is critical; moreover, this versatile organelle has developed protective measures of dysfunctional mitochondria elimination and repair, involving mitochondrial dynamics [75]. These dynamic processes include mitochondrial fusion, fission, trafficking, and clearance through selective autophagy of this organelle, termed mitophagy [18]. Mitochondrial fusion provides macromolecular and Ca^2+^ exchange between neighboring mitochondria and complements mtDNA. The process restores functional proteins and non-damaged mtDNA to dysfunctional mitochondria, therefore, decreasing the occurrence of mitophagy. Chief fusion machinery includes three GTPases: the mitofusins 1 and 2 (Mfn1, Mfn2) mediate OM fusion; and the third GTPase, the optic dominant atrophy (OPA1), mediates IM fusion [76]. Mitochondrial fission segregates dysfunctional parts of the mitochondria; these fragments can subsequently be degraded through lysosomal-related pathways via mitophagy. This fragmented structure allows for more efficient engulfment by autolysosome machinery, while interconnected tubular mitochondria are protected [18]. The master fission proteins include the dynamin-related GTPase protein 1 (Drp1), the mammalian Drp1 homolog dynamin like protein 1 (DLP1), and mitochondrial fission 1 protein (Fis1) [21]. 

### 2.3. The Defenses of Mitochondria Dysfunction: Mitochondrial Protein Homeostasis and Mitochondrial-Derived Vesicles 

The major players of mitochondrial protein homeostasis (proteostasis) include mitochondrial chaperones, such as mitochondrial 70 kilodalton heat shock proteins (mtHsp70), mitochondrial heat shock protein (Hsp60), tumor necrosis factor receptor-associated protein 1/Hsp90 (Trap1), and mortalin (HSPA9). These chaperones assist in protein folding/re-folding and prevention of aggregate formation [77]. Damaged or terminally misfolded proteins within the matrix are degraded by mitochondria-localized proteases such as LON, ClpXP and m-AAA proteases. The proteins in the OM are ubiquitinated by E3 ligase MARCH5 and targeted to the 26S proteasome [20]. When unfolded or misfolded proteins in the mitochondria go beyond the capacity of chaperone proteins, the importation of mitochondrial proteins is hindered, initiating the mitochondrial unfolded protein response (UPR^mt^) by eliciting the increased transcription of nuclear genes for mitochondrial chaperones and proteases to promote the recovery of organellar proteostasis [78]. A regulator of UPR^mt^, the stress-activated transcription factor ATFS-1 is normally imported rapidly into healthy mitochondria and degraded. However, during mitochondrial misfolded protein accumulation and impaired import of mitochondrial-targeted proteins due to decreased Δψm, a percentage of the ATFS-1 translocate to the nucleus where they upregulate mitochondrial chaperones and proteases, downregulate the TCA-cycle and OXPHOS transcripts, and increase glycolysis and amino acid catabolism. When combined, the load on damaged mitochondria is decreased via substitute intracellular energy generation through other pathways and the clearing up of unwanted mitochondrial and cellular proteins. If the proteostasis quality control is insufficient for stress homeostasis, mitochondrial-derived vesicles (MDVs) and mitophagy target partial or whole mitochondria for degradation through the lysosomal system [79]. The cargo selective MDVs bud from the organelle independent from the fission machinery and eliminate larger amounts of misfolded proteins and ROS at the lysosome in a mitophagy-independent manner [80]. The PD-linked PINK1/parkin pathway has been found to be implicated in both of these mechanisms, to the point when stress adaptation attempts may not achieve homeostasis, quality control at the cellular level is carried out through MOMP activation of intrinsic apoptosis [81]. 

### 2.4. The Clearance of Damaged Mitochondria: Mitophagy and Underlying Autophagic Mechanisms

Mitophagy, the selective degradation of mitochondria via autophagy (self-eating), is a key process for maintaining mitochondrial homeostasis. Mitochondrial turnover via this mechanism is essential for maintaining neuronal health since autophagy sequesters, degrades, and recycles cellular material [74]. Abnormal mitochondrial autophagy accompanies neurodegeneration. Several PD-related proteins are known to participate in the regulation of mitophagy, and the phosphatase and tensin homolog (PTEN)-induced putative kinase 1 (PINK1) and parkin pathway regulates ubiquitin (Ub)-dependent mitophagy. PINK1 localizes to the mitochondria under normal conditions and is rapidly translocated to the IM to be cleaved and inactivated by IM protease presenilins-associated rhomboid-like protein (PARL). PINK1 fragments are then released into the cytosol to be degraded via the N-end rule pathway. When the damaged mitochondrion become depolarized, PINK1 is unable to enter the mitochondrion, resulting in PINK1 accumulation on the OM. Parkin is normally inactivated cytosolic proteins. PINK1 localization to mitochondrial OM activates parkin by phosphorylation and recruits parkin to the OM where the protein assembles a ubiquitin chain on OM proteins. Parkin subsequently recruits ubiquitin-binding mitophagy receptors (including p62) to the mitochondrion, which promotes capture by light chain 3 (LC3-II)-positive phagophores. The enclosed mitochondrion is then trafficked to the lysosome for further degradation. 

Autophagy is a cytoplasmic catabolic process that prevents the build-up of misfolded proteins, removes damaged organelles, and provides the cell and organism with bioenergetic substrates necessary for survival [82,83]. Three types of autophagy have been reported. The first type is microautophagy, responsible for the direct invagination and degradation of soluble or particulate cytoplasmic contents into the lysosomes [84,85]. The second type, chaperone-mediated autophagy (CMA), involves the chaperone heat shock cognate 71 kDa protein (Hsc70), which discriminates KFERQ-like-motif-bearing proteins and its delivery to the lysosome. The lysosome-associated membrane protein type 2A (LAMP2A) assists with protein-lysosomal docking, internalization, and final degradation, and is involved in the clearance of damaged proteins including α-syn [86]. The third type is macroautophagy, sometimes referred to as autophagy. In this process, waste products are enclosed within double membrane structures and brought to the lysosome for degradation. The mechanism involves initiation, nucleation, elongation, lysosomal fusion, and degradation. Macroautophagy (hereafter “autophagy”) originates from conditions of stress including starvation, oxidative stress, protein aggregation, and ER stress [87]. The common target of different stress signaling pathways is the Unc-51-like kinase 1 (ULK1) complex, the initiation of which phosphorylates the class III PI3K (PI3KC3) Complex I [88]. The PI3KC3-C1 then assists phosphatidylinositol 3-phosphate (PI(3)P) production at the isolation membrane of the ER, from which transient double-membraned phagophores are formed [89]. Downstream proteins and complexes work to enhance autophagy-related proteins ATG8-family-protein (ATG8s) binding to the phosphatidylethanolamine (PE) on the membrane. ATG8s (including the microtubule-associated protein light chain 3 (LC3) proteins) assist in nucleation, recruiting LC3-interacting-region(LIR)-motif-bearing autophagy factors, and selectively sequestering specifically tagged cargo via LIR cargo receptors [90]. While ATG8s also facilitate elongation and closure of the phagophore membrane to form the autophagosome, the insertion of lipidated-ATG8s to autophagosome membrane drives autophagosome maturation [91]. The lipidated family member LC3-II bound on the autophagic membrane is a characteristic signature of autophagic activation [92]. Elongation of autophagosomal membranes is supplied by ATG9-containing vesicles bringing lipid bilayers from the plasma membrane, the mitochondria, recycling endosomes, and the Golgi complex. Autophagosomes are trafficked to and fuse with a lysosome, forming an autolysosome and trapped cargo is subsequently degraded [93]. During starvation, the AMP-activated protein kinase (AMPK) phosphorylates tuberous sclerosis complex 2 (TSC2) and inactivates a negative regulator of autophagy, the mammalian target of rapamycin complex (mTORC), triggering autophagy and promoting survival [94]. Another regulator of autophagic proteins is the pro-autophagic protein Beclin 1, a part of the PI3KC3 complex. Beclin 1 is activated once phosphorylated by kinases including AMPK to stimulate autophagy. Autophagy may be a survival mechanism for cells under acute stress. In the event of autophagic vesicle accumulation, impaired autophagic flux autophagy may become detrimental, leading to apoptosis. Mitophagy is of especial importance in high energy demand neurons for its selective autophagic elimination of potentially harmful damaged mitochondria and the maintenance of mitochondrial homeostasis [13]. 

### 2.5. Mitochondrial ETC Dysfunction and PD

In the initial report of an association between mitochondria and PD, scientists discovered a link with the usage of a recreational opioid analog with contaminate MPTP, resulting in PD-like motor symptoms associated with SN degeneration. Experiments showed that its metabolite MPP^+^, a mitochondrial respiratory chain Complex I inhibitor, is selectively imported to DA neurons after passing the blood-brain barrier [95]. Lewy body pathology and DA neuronal loss were noted upon autopsy [96]. Studies have suggested inhibitor binding to mitochondrial Complex I as a molecular initiating event (MIE) for the adverse outcome (AO), namely Parkinsonian motor deficits. Key events within this process include mitochondrial dysfunction, impaired proteostasis, and eventual degeneration of DA neurons within the SN [97]. With the bridge between mitochondrial ETC complex dysfunction and PD established, links between other environmental mitochondrial toxin exposures and parkinsonism were subsequently investigated. Pesticides, herbicides, insecticides such as paraquat, rotenone, and maneb have been shown to cause PD through ETC interference and are now frequently used for PD toxin models [43,98]. Paraquat and rotenone are notorious Complex I inhibitors, and the manganese ethylene-bis-dithiocarbamate (Mn-EBDC) containing maneb inhibits Complex III. A multitude of studies has explored the pathogenic mechanisms behind mitochondrial involvement in PD through rotenone PD models which reveal these toxins increase ROS production, decrease mitochondrial membrane potential, induce apoptosis, and hinder autophagic flux [99,100]. During the past two decades, genetic investigations have revealed numerous mechanistic findings, notably the involvement of mitochondria-related pathways in PD [101]. 

### 2.6. Genetic Links of Mitochondria to PD 

The role of genetics in the etiology of PD has been debated, as less than 15% of PD patients have a family history, and no more than 10% have Mendelian inheritance [102]. However, these hereditary forms of PD have provided crucial clues to the underlying neuropathology of PD, with large genome-wide association studies (GWAS) have confirmed that these genes may have effects in sporadic PD. Many of the genes responsible for familial PD have strong links to mitochondrial function. These include the autosomal recessive familial genes PRKN (PARK2), PINK1 (PARK6), Daisuke-Junko-1 (DJ-1/PARK7), ATPase type13A2 (ATP13A2, PARK9), and F-box only protein 7 (FBXO7/PARK15). The autosomal dominant PD genes involved include the SNCA (PARK1), Leucine rich repeat Kinase 2 (LRRK2/PARK8), and vacuolar protein sorting 35 (VPS35/PARK17) (Table 1). 

Loss of function mutations in PINK1 and parkin genes have been associated with juvenile PD, with mutated parkin protein recording the greatest frequency. The PINK1 protein is a kinase localized on mitochondria and parkin, a ubiquitin(Ub)-E3 ligase, and the PINK1-dependent activation of parkin plays a critical role in the process of Ub-dependent mitophagy, as previously stated [103]. PINK1 and parkin are also shown to modify a wide range of substrate proteins and mediate their clearance. This includes targeting fusion proteins Mfn-1 and Mfn-2 to prevent the re-fusion of damaged mitochondria back into the healthy network. These Mfns are eliminated through mitochondria-assisted degradation via the 26S proteasome. Due to inhibited fusion, further fragmentation of damaged mitochondria is induced, possibly leading to the formation of MDVs for localized quality control. Damaged mitochondrial transport along axons is also hindered by the degradation of Miro, a mitochondrial transportation protein, through PINK1 and parkin-dependent proteasome pathways. This inhibition of the mitochondrial movement may prevent the spreading of dysfunctional organelles along neurons and could be a quarantine step prior to mitophagy [104,105]. Other roles of PINK1 and parkin for mitochondrial health include targeting the transcriptional repressor parkin interacting substrate (PARIS) for proteasomal degradation, promoting the transcriptional coactivator PGC-1α for mitochondrial biogenesis. Loss of parkin function could impair mitochondrial biogenesis and may lead to cell death [106]. The anti-apoptosis role of parkin has been reported, as the ligase oppose pro-apoptotic Bax translocation to the mitochondria and therefore inhibit subsequent cytochrome c release [107]. The recessive PD gene FBXO7 encodes a component of the E3 Ub ligase, the reduced expression of which leads to deficient parkin translocation to damaged mitochondria, decreased Mfn ubiqutination, and impaired mitophagy [108]. The loss of function mutations of the DJ-1 gene is associated with recessive early-onset familial PD and late-onset sporadic PD [109]. The gene encodes the 189-amino-acid DJ-1 protein, with studies having demonstrated greater DJ-1 oxidative damage and elevated DJ-1 protein levels in postmortem brains of sporadic PD patients [110]. This ubiquitous pleiotropic protein has been proposed to play a protective role in multiple functions, including redox-sensitive esterase, chaperone for α-syn, protease, transcriptional regulator, the regulator of pro-apoptotic Bax, the regulator of tyrosine hydroxylase in dopamine synthesis, and regulator of the 20S proteasome [111]. DJ-1 has a highly oxidative-stress sensitive cysteine residue at position 106 (Cys-106), which induces DJ-1 localization to the depolarized mitochondria to regulate antioxidant defense and maintain Complex I activity [112,113,114]. Knockdown of DJ-1 has resulted in impaired Complex I function, reduced Δψm, fragmented mitochondria, impaired Ca^2+^ dynamics, with increased oxidative damage and mitochondrial dysfunction [115,116]. Sharma et al. demonstrated that reduced DJ-1 activity due to oxidative stress may lead to α-syn aggregation [117]. Meanwhile, deficiency of another PD recessive gene, ATP13A2, causes fragmentation of the mitochondria network, increased ROS production, and decreased autophagic events [118]. The SNCA gene is the first familial autosomal dominant PD-associated gene encoding the α-syn protein, which is proposed to function in dopamine release regulation, fibrillization of the protein tau, inhibition of tumor suppressor protein p53, and inhibition of other pro-apoptotic genes [119]. A fraction of cellular α-syn selectively localizes to the mitochondria upon specific stimuli, and the protein directly influences cytochrome c release, Ca^2+^ homeostasis, Δψm mediation, and OXPHOS efficiency [120,121,122]. The mutation of the SNCA gene causes Complex I inhibition and mitochondria fragmentation, which could be rescued by co-expression of PINK1, parkin, and DJ-1 [123]. Studies have also shown α-syn binding to the translocase of the outer membrane (TOM) protein, thereby impairing mitochondrial protein importation and further affecting the PINK1 and parkin pathways [124]. The deficiency of LRRK2 has also been shown to cause α-syn accumulation and autophagic stress [125,126]. The major autosomal dominant PD-related gene LRRK2 is the most frequent genetic cause of familial and sporadic PD. The gene product is a 286-kDa ROCO family protein with a kinase and a GTPase domain [127]. LRRK2 functions in cytoskeletal maintenance, vesicle trafficking, autophagy protein degradation, and the immune system [126]. LRRK2 deficiency leads to reduced Δψm, dysregulation of mitochondria fusion and fission, higher levels of mtDNA damage, and reduced cell survival. The third autosomal dominant PD gene is the VPS35. The protein is part of a retromer complex that plays an important part in endosome-Golgi-plasma membrane protein transport, and also plays a role in MDVs, shuttling cargo from mitochondria to peroxisomes or lysosomes [128]. Through MDVs, VPS35 mediates mitochondrial fission complex DLP1 recycling. As such, Wang et al. have demonstrated that PD-associated VPS35 mutation causes increased DLP1 turnover and excessive fission which further leads to mitochondrial dysfunction and neuronal loss [129]. 

### 2.7. Mitochondrion as a Therapeutic Target for PD 

Since mitochondrial dysfunction takes center stage for PD pathogenesis, approaches aimed at modulating mitochondrial function as a therapeutic target for PD treatment include mitochondrial biogenesis induction, enhancing mitochondrial quality control, preventing mitochondrial involved cell death, and altering mitochondrial signaling and metabolic pathways [130]. Promising candidate drugs and supplements include nicotinamide adenine dinucleotide (NAD^+^) replenishment with nicotinamide mononucleotide (NMN), mitochondrially targeted antioxidants MitoQ and SS-31, free radical scavenger Coenzyme Q10, and mPTP inhibitors for blocking apoptosis creatine and cyclosporin A [131]. NAD^+^ is a cofactor and electron carrier in the ETC, whose depletion leads to disrupted mitochondrial ETC ATP production, the elevation of protein lysine acylation resulting in damaged and misfolded proteins accumulation, and mPTP formation leading to cell death [132,133,134]. Other compounds with antioxidative qualities include resveratrol which has also been demonstrated to affect mitochondrial morphology, autophagic flux, and mitochondrial biogenesis enhancing effects in PD cellular models [101,135]. Additional pharmacological interventions aimed at transcription factors and their co-activators involving in mitochondrial biogenesis have been demonstrated, such as nuclear respiratory factor 1 and 2 (NRF1, NRF2), mitochondrial transcription factor A (TFAM), and peroxisome proliferator-activated receptor gamma (PPARγ) co-activator 1 α (PGC1α) which affect mtDNA replication, expression, and coordinate organelle biogenesis in response to metabolic challenges [136,137]. An interesting study by Lin et al. showed healthy mitochondria transfer from Wharton’s jelly mesenchymal stem cells (WJMSCs) to cells harboring a mtDNA defect in rotenone-stressed mitochondrial myopathy, encephalomyopathy, lactic acidosis, and stroke-like episodes (MELAS) patient cells, demonstrating that healthy mitochondria transfer may be a potential treatment avenue in rescuing dysfunctional mitochondria [138]. Though the mitochondrial strategies have not yet proven rewarding on humans, there are still promises of its potential usage in delaying PD disease progression in the future. 

## 3. Alpha-Synuclein Accumulation and PD

### 3.1. Alpha-Synuclein: The Main Component Protein of Lewy Body

As people age, unfolded proteins and aggregates progressively accumulate in specific regions of the brain, leading to proteotoxic neurodegenerative diseases, with α-syn accumulation in the case of PD [139]. Aggregated α-syn are the main components of cytosolic eosinophilic Lewy body inclusions, which are found in afflicted central and peripheral nervous systems of PD patients, most abundant in the SNpc where selective DA neuron loss is greatest [140]. Of note, α-syn protein is encoded by the SNCA gene, the first gene associated with familial PD. The protein is mainly localized at the presynaptic terminal where α-syn promotes membrane curvature and assembly of the soluble N-ethylmaleimide-sensitive factor attachment protein receptor (SNARE) complex, a mediator for vesicle fusion with target membranes [141]. Composed of 140 amino acids, α-syn contributes to synaptic trafficking, vesicle budding, and vesicle recycling; while in the case of DA neurons, it mediates dopamine synthesis, storage, and release [142,143]. In addition, α-syn interacts with membrane lipids on mitochondria and ER, and play a part in Ca^2+^ homeostasis. The α-syn protein is divided into three major domains: the amphipathic N-terminus (1-60), the hydrophobic central non-amyloid component (NAC) (61-95), and the acidic C-terminus (96-140). The N-terminal is characterized by lipid-binding α-helixes formed by seven repeats of 11 amino acids containing the conserved KTKEGV hexameric motif. Various point mutations at coordinates of the N-terminal (A53E/T, A30P, E46K H50Q, and G51D) are likely involved in membrane lipid interactions and are linked to dominant familial parkinsonism and PD [144]. The NAC is the core of α-syn and deletions in this region are essential for misfolding and aggregation of α-syn into fibrils. The C-terminus is a charged unstructured tail important for the prevention of α-syn aggregation and also helps regulate synaptic vesicles [145]. Multimeric conformations of physiological α-syn co-exist within the cell, including non-toxic soluble unfolded monomers and, in large part, the aggregation-resistant tetramers [146]. In high concentrations, α-syn has the propensity to convert into pathological oligomers and self-aggregates into higher-order fibrils with amyloid cross-beta conformation, which are then deposited into Lewy bodies and Lewy neurites in affected neurons [147]. Oligomers are aggregated α-syn that have not acquired a fibrillary conformation and encompass a wide spectrum of molecular weights and beta-sheet content. Larger oligomers are more stable, process high seeding properties, and are highly toxic [148,149]. These oligomers and amyloid fibrils notably affect mitochondrial function, ER–Golgi trafficking, protein degradation, and synaptic transmission, which induce neurodegeneration. Zhang et al. have suggested nigral neuronal damage releases aggregated α-syn into the substantia nigra, activating microglia and producing subsequent pro-inflammatory mediators and ROS [150]. Oxidative stress, toxins, and interaction with oxidized dopamine further increase the propensity of α-syn to aggregate and accumulate. Research has also demonstrated excess α-syn to inhibit dopamine biosynthesis through interaction with tyrosine hydroxylase and impair dopamine uptake via dopamine transporter [142]. Therefore, DA neurons are focal aggregation sites due in part to the numerous synapses these neurons possess as well as the increased oxidative stress caused by dopamine production. The pathological course of α-syn involves the spread of disease; as such, reports have shown that α-syn oligomers and fibrils, as well as monomers, can transfer between cells and propagate the disease to other brain regions - the prion-like theory [151,152]. Once inside host cells, α-syn aggregates undergo nucleation, aggregation, and propagation [153]. The spreading mechanisms involved are multiple and can occur via endocytosis, direct penetration, trans-synaptic transmission, or membrane receptors. Furthermore, the pathogenic roles of α-syn are supported by genetic data, as multiplications of SNCA, and N-terminal point mutations result in dominant familial PD [144]. SNCA gene overexpression results in the formation of intracellular insoluble α-syn, with other underlying causes of unfavorable α-syn accumulation including post-translational modulation, and impaired α-syn degradation which consequently leads to neuron degeneration. There are two major proteolytic pathways implicated in α-syn processing, the ubiquitin-dependent and -independent proteasome systems, and the macroautophagy and CMA autophagy-endo-lysosomal systems [16,154,155]. The monomeric form of α-syn are predominantly degraded by CMA, and ser-129 phosphorylation on α-syn leads to degradation via the proteasome system [155]. Numerous PD-linked mutations or failed proteases are linked to a deficient ubiquitin-proteasome system and dysfunctional CMA, such as the A53T and A30P SNCA point mutations, and some autophagy-lysosomal related genes [156]. 

### 3.2. The Translocation of Alpha-Synuclein to the Mitochondria and Nucleus 

The relationship between mitochondria and α-syn is one of interconnection; more specifically, α-syn overexpression or genetic mutation causes dysfunction of mitochondria, mitochondrial fragmentation, mitochondrial membrane shaping inhibition, mitochondrial axonal transport decrease, and impaired mitochondrial-ER Ca^2+^ exchange at the mitochondria-associated membrane [17,157]. In post mortem PD brains and animal models, overexpressed or mutant α-syn accumulate in the ER, thus impairing protein folding and evoking ER stress. A53T α-syn plays a role in impairing both mitochondrial and ER, thereby generating an environment of oxidative and ER dysfunction, and consequent neuronal death. Stressed ER transfer excess Ca^2+^ to the mitochondria, which then generate even more ROS [158]. ER and mitochondrial stress both activate antioxidative systems, which in turn activate the UPR^mt^, helping to reestablish the redox states of both organelles [16]. Different immunogold electron microscopy studies have reported α-syn localization to the mitochondrial OM, IMS, and IM [17]. Research has indicated that A53T mutated α-syn targeting to mitochondrial OM induces OM curvature changes and surrounding mitochondrial fragmentation, suggesting α-syn directly binds to the mitochondrial OM, with such interaction interrupting mitochondrial morphology [124]. α-syn accumulation has also been identified in mitochondria in model cells, different regions of mouse brains, and also post-mortem brains of PD patients [18]. The N-terminus domain of α-syn has been observed to mimic mitochondrial targeting sequence properties and may promote anchoring of the protein to mitochondrial membranes [159]. Distinct pathogenic variants that contain a single amino acid substitution in the first 32 amino acids of the N-terminus display different affinities for membranes and are fundamental for mitochondrial localization, suggesting that aside from the role in protein aggregation, the N-terminus could impact α-syn association to intracellular membranes, and thus α-syn subcellular localization. This notion is supported by the observation that A53T α-syn variant is highly enriched in mitochondria as compared to A30T variants under general and oxidative-stressed conditions; the E46K mutant protein show higher affinity for vesicles containing more negatively charged lipids [159]. Although the specific mechanisms of α-syn importation into mitochondria are not fully understood, it has been shown to involve Δψm dependency, the TOM complex, and the voltage-dependent anion channels (VDAC/ mitochondrial porins). The import of α-syn into mitochondria impairs a broad range of mitochondrial functions, and when α-syn further accumulate within the mitochondria, translocase of the inner and outer membrane (TIM/TOM) complex assembly process is disrupted, subsequently hindering mitochondrial protein import from the cytosol [17,160]. The mitochondrial translocation of α-syn is enhanced by cellular stress and low intracellular pH [161]. Interestingly, Ludtmann et al. demonstrated α-syn function in the mitochondrial matrix, suggesting that a pool of physiological α-syn increases ATP synthase activity through directly binding to its α subunit, thereby ensuring mitochondrial health and proper ATP fueling for synaptic function [124,162]. Furthermore, α-syn nuclear localization has been observed, and α-syn binding to DNA upon increased oxidative stress presents the genetic modulatory functions of the protein. The precise influence of α-syn on the nucleus remains controversial, with some studies reporting that nuclear α-syn downregulated major DNA repair and cell cycle-related genes, and others reporting the protective effects on mitochondrial biogenesis and histone hyperacetylation [163]. Pinho et al. have noted phosphorylation at specific α-syn sites and nuclear presence of different α-syn species affecting the role of α-syn on gene expression and neurotoxicity [164]. 

### 3.3. Future Prospect: Alpha-Synuclein as a Treatment Target for PD 

Due to the numerous toxic effects of α-syn aggregates, strategies to reduce α-syn accumulation have been developed, including decreasing α-syn synthesis through use of small interfering RNA (siRNA) or microRNA (miRNA), and activating clearance mechanisms such as autophagy, the proteasome, neurosin, matrix metalloproteinase 9 (MMP9), and heat shock proteins [165]. In in vitro and ex vivo models, Loria et al. demonstrated efficiency differences of α-syn aggregation transfer between neurons and astrocytes. They also exhibited that α-syn puncta were mainly localized within the lysosomal compartment of the recipient cell and that differently from neurons, astrocytes are able to degrade α-syn fibrils efficiently. This data indicates that astrocytes are able to trap and clear pathological α-syn deposits in PD [166]. In addition, anti-aggregating, antioxidant or post-translational modification approaches, and immunotherapy may be used to block transmission and oligomer formation and thus α-syn aggregation. Of note, since mitochondria are the major source of intracellular ROS under normal physiological conditions, antioxidants against mitochondria-related oxidative stress can be a potential target for the prevention of ROS-induced α-syn protein denaturing and aggregation [158]. As recently reported, overexpression of the lysosomal transcription factor TFEB in rats, expressing α-synuclein decreases α-synuclein oligomer levels and prevents lysosomal dysfunction decline and neurodegeneration; therefore, augmentation of endo-lysosomal function to enhance damaged protein and organelle turnover can potentially protect neurons from α-syn pathology with excessive damaged protein accumulation [165]. 

## 4. Lysosomal Disorders and Parkinson’s Disease: Interrelationship with Mitochondrial Dysfunction and Alpha-synuclein Aggregation 

### 4.1. The Biological Function of Lysosome beyond Protein Catabolism 

Lysosomes are membrane-enclosed cytoplasmic organelles in the endomembrane system of animal cells and harbor a diverse array of hydrolases. As the degradative endpoint for intracellular and exogenous biomacromolecules, these cellular quality controllers are highly dynamic in intracellular trafficking and distribution, and they continuously fuse and fission with each other as well as other organelles, including late endosomes, phagosomes, and autophagosomes for autophagy [167]. According to hydrolases requirements, vacuolar ATPase (V-ATPase) pumps protons into the lysosome to maintain a pH of 4.5–5.0. Lysosomes are also recognized as sophisticated signaling centers that are able to interface physically and functionally with other organelles to govern cell growth, division, and differentiation [168]. The physical membrane contact with organelles such as mitochondria and the ER aid in mitochondrial fission and may participate in metabolic regulatory roles through nutrient and metabolite sharing [169]. Involved in critical cellular anabolic-catabolic function, the lysosomal function is under strict signaling regulations, including recruitment and activation of the master growth regulator, mammalian target of rapamycin complex 1 (mTORC1) protein kinase [170]. mTORC1 is activated at the lysosomal surface under the dual presence of nutrients and growth factors, and its activation triggers downstream anabolic pathways, directing cells toward growth. During starvation conditions, mTORC1 enhances autophagy by disinhibition of the autophagosome initiators Atg1 kinase and the Atg13 protein, and via the MiT/TFE basic leucine zipper transcription factors, which coordinate many lysosomal target gene expressions, increasing autophagosome numbers, lysosome biogenesis, and lipid catabolism [171,172,173]. Lysosome lumens also serve as storage sites where amino acids, phosphate, ions, and intermediate metabolites can be selectively transported and retained [174]. Moreover, faulty mutations of lysosomal genes lead to a spectrum of metabolic diseases known as lysosomal storage disorders, and defective execution of lysosome growth and catabolic programs have notably been implicated in neurodegeneration, cancer, and age-related diseases [175]. 

### 4.2. Link between Parkinson’s Disease and Glucocerebrosidase Gene Mutations 

The clinical correlation between PD and lysosomal dysfunction was initially observed when a small group of patients with GD, a lysosomal storage disease, presented with parkinsonian symptoms [176,177]. GD is caused by the loss of function in the GBA1 gene which encodes the lysosomal hydroxylase, β-glucocerebrosidase (GCase) [178]. In 2003, Sidransky et al. reported on a cohort of 17 GD patients who developed PD symptoms at a mean age of 48, including patients of different ethnic origins [179]. Autopsies were performed in four of the patients and α-syn-positive Lewy body pathology was observed in the GD-vulnerable hippocampal layers CA2-4 [180,181,182]. Meanwhile, Wong et al. studied 7 GD patients with GBA-associated parkinsonism and found α-syn-immunoreactive cortical-type and brain-stem-type Lewy bodies, and Lewy neurites [183]. Subsequent studies have revealed that some of the probands have a positive family history of parkinsonism; specifically, while concurrent GD and PD is rare, the more frequent phenomenon of non-GD diseased family members with early developing PD symptoms has been noted, demonstrating higher PD risk in carriers [184]. In other studies, a higher incidence of the GBA1 mutation was noted in pathologically-confirmed, idiopathic PD brains [179]. GBA1 mutation frequency has shown heterozygous GBA mutations in 10.7%–31.3% of Ashkenazi Jewish patients with PD, and 1.3%–9.4% in other ethnic origins worldwide [185,186,187]. As for the role of GBA1 mutations in PD, there has been a hypothesis for the gain of function, loss of function, and prion-like mechanism [188,189]. Toxic gain of function by GBA1 mutation suggests aberrant misfolded-mutant GCase interaction with cellular protein homeostasis, including the quality control machinery in the ER [190]. The chronic stress of ER combined with ROS accumulation may further lead to widespread cellular dysfunction [191]. Functional loss of GCase activity due to GBA1 mutations have been shown to decrease lysosomal proteolysis, disrupt the ubiquitin-proteasome pathway, and interfere with autophagic processes, thereby further inducing α-syn accumulation and neuron toxicity in human midbrain neuron cultures [192]. Prion hypothesis and the possible combination of the second hit hypothesis suggests that a cell-to-cell α-syn transmission through a lipid-rich environment may occur during pathological development [193]. Jakob et al. suggested lysosomal importance in the processing and spreading of aggregated α-syn between neurons in their study of cultured human SH-SY5Y cells, and notably observed that most α-syn co-localized with the lysosomal/endosomal system, both pre- and post-synaptically [24]. Alternatively, α-syn accumulation disrupts GCase trafficking to lysosomes and decreases GCase activity, which may further exacerbate the vicious cycle of protein misfolding underlying GBA-associated PD Lewy body formation [194,195]. Supporting this, enhancing GCase activity has been shown to decrease α-syn accumulation and salvage lysosomal, mitochondrial, and neuronal dysfunction [196]. Further investigation into the mechanisms that contribute to GBA-associated parkinsonism and to identify other risk factors that may function concurrently are required. 

### 4.3. PD Susceptible Gene Mutation and Lysosomal Dysfunction 

Several partially-penetrant mutations of PD converge on cellular clearance pathways, this has been observed in several mechanistic studies that have linked lysosomal dysfunction to defective mitochondrial clearance and α-syn toxicity [37]. As such, deletion of mitochondrial proteins PINK1, OPA1, and apoptosis-inducing factor (AIF) in a mouse cortical neuron model has resulted in defective lysosomal acidification, decreased lysosomal activity, and large cytoplasmic late-endosome-marker-positive vacuole formation; while further addition of antioxidants to these mitochondria dysfunctional neurons exhibited improvement of decreased lysosomal activity [197,198]. In addition to the critical roles in the well-known mitophagy and proteasome degradation pathways [167,199,200], PINK1/parkin has recently been found to be involved in rapid lysosomal targeting of oxidized mitochondrial proteins via MDVs [201]. McLelland et al. noted that in response to oxidative stress, parkin induces PINK1-dependent mitochondrial vesicles formation which incorporates oxidized cargo and shuttles them to late endosomes for protein degradation [202]. Parkin has also been shown to modulate the endosomal structure and function and has displayed interaction with VPS35 to affect MDVs formation [203]. This vesicular trafficking pathway is PINK1/parkin-dependent, autophagy-independent, and lysosome targeted, hence preserving the integrity of mitochondria. Using PD in vivo and in vitro models, Xu et al. demonstrated that α-syn overexpression activated CMA by elevation of LAMP2A, and that DJ-1 knockout/down suppressed LAMP2A upregulation as well as accelerated LAMP2A degradation in the lysosomes, further inhibiting α-syn degradation [204]. In 2015, Oren et al. also demonstrated rigorous regulation of DJ-1 on the major proteolytic machinery, the 20S proteasome, under the oxidizing environment [205]. The 20S proteasome eliminates oxidative-damaged protein, keeps high maintenance of native intrinsic unstructured proteins for regulatory and signaling events, and plays a part in ubiquitin-independent autophagosome-lysosome fusion [206,207,208]. Lysosomal association of the most frequent PD-associated gene LRRK2, accounting for 40% of genetic cases of PD, has also been connected, depicting interaction with many proteins in the endo-lysosome compartment and the role played in autophagosome formation, lysosome maturation, and lysosome trafficking [209]. Meanwhile, LRRK2 mutations have been shown to interfere with mitochondria fission factor DLP1, causing mitochondrial dynamic imbalance, disturbing mitochondrial quality control. These effects combined lead to the eventual accumulation of damaged mitochondria [210,211]. 

### 4.4. Conversely Lysosomal Genes Mutations Link to Parkinson’s Disease

A causal relationship has been identified between heterozygous mutations in the lysosomal P5-type transport ATPase, ATP13A2 gene, which cause an autosomal recessive form of juvenile-onset parkinsonism [212]. The function of ATP13A2 is elusive and its transported substrate still to be identified; however, evidence has shown the role of ATP13A2 in Zn^2+^, Fe^3+^ and Mn^2+^ homeostasis [213,214]. Reports by Grünewald et al. and Gusdon et al. have shown that depletion of the ATP13A2 gene is associated with decreased mitochondrial turnover, impaired lysosomal acidification, proteolytic processing of lysosomal enzymes, and diminished lysosomal-mediated clearance of autophagosomes [215,216]. Another well-known PD-associated lysosomal protein, VPS35, mediates MDVs transport between mitochondria and other cellular compartments and is associated with various neurodegenerative disorders including both PD and Alzheimer’s disease [217,218]. Pathogenic D620N mutation in VPS35 disrupts both endosomal protein trafficking and the other main target, the mitochondria [129]. In addition, investigations of mouse cultured neurons have shown depletion of VPS35 reduces Mfn2 stabilization and impedes mitochondrial fusion, resulting in mitochondrial fragmentation [219]. Drosophila experiments revealed VPS35 part in the PINK1-parkin pathway, in which overexpression of VPS35 salvaged parkin but not PINK1 phenotypes [220]. Tang et al. show in their research specific deletion of the VPS35 gene in mice DA neurons to cause neuronal loss and α-syn accumulation [219]. Identification of PD related genes through GWAS include a signal at chromosome 4p16.3, and one variant was discovered in the human transmembrane protein 175 (TMEM175) [221]. TMEM175 is a lysosomal K^+^ channel transmembrane protein, whose deficiency has been found to impair lysosomal acidification, causing mitochondrial dysfunction, influencing α-syn phosphorylation, and impairing autophagy [222]. Also noted through the GWAS study, sterol regulatory element-binding transcription factor 1 (SREBF1) links lipogenesis to PD [223,224]. SREBF1 is a transcriptional activator imperative for the regulation of lysosomal lipid and cholesterol accumulation [225]. Of note, knockdown of the SREBF1 has been shown to block the translocation of parkin to the mitochondria, consequently decreasing mitophagy [226]. The endo-lysosomal pathways, mitochondrial dysfunction, and α-syn aggregation have been extensively documented to be involved in the pathogenesis of PD. Genetic and mechanistic investigations have revealed the intricate interplay of how dysregulation of one system impacts the other two. Whether one of the three factors is dominant or whether they develop concomitantly in the process of PD neurodegeneration remains unclear, however strong associations between the three factors and the disease have been established, akin to traffic jams at overcrowded crossroads (Table 1).

## 5. Conclusions

The pathogenesis of PD is a multifactorial process, with the primary contributors known to be age, environment, and genetic factors. Although familial parkinsonism makes up less than 10% of adult parkinsonism, the findings generated from these genetic function studies have greatly enhanced the understanding of the processes involved in neuron degeneration. These include mitochondrial dysfunction, disruption of network integrity, and α-syn accumulation. Meanwhile, the functions of the proteasome and endo-lysosomal pathways in cellular degradation are also involved. Mitochondrial dysfunctions, endo-lysosomal disruptions, and α-syn aggregation mutually interact within neurons, while α-syn prion-like propagation may also be associated with PD in an inter-neuronal manner. Both mitochondria and endo-lysosomal dysfunction contribute to the development of α-syn pathology, however, the specific organelle playing the most important role might be decided by individual genetic and environmental factors, based on the highly variable clinical presentations among PD patients. Most likely, a vicious cycle may develop once one system becomes dysfunctional. Although successful therapies to control PD progression have yet to be identified, further elucidation of the precise molecular mechanisms involved in the pathogenesis of PD may provide guidance for the development of future therapeutic targets to treat this complex disease. 

## Figures and Tables

**Table 1:** Selected PD associated genes and their mitochondria, endo-lysosomal system, and α-synuclein pathology phenotype.

Gene /Mode of Inheritance	Locus Symbol	Main Function	Mitochondrial Dysfunction	Endo-Lysosomal Dysfunction	α-Synuclein Pathology	Reference
*SNCA* (α-*synuclein)* AD	*PARK1*	Synaptic vesicle recycling	+	+	+ PD patients	[165,227]
*PRKN (Parkin)* AR	*PARK2*	E3 ubiquitin ligase (Mitophagy)	+	+	+ PD patients	[47,228,229,230]
*PINK1* AR	*PARK6*	Kinase (Mitophagy)	+	+	+ PD patients	[79,231,232,233]
*DJ-1* AR	*PARK7*	Antioxidant, α-syn chaperone, protease, transcription factor (Mitophagy)	+	+	+ PD patients	[234]
*LRRK2* AD	*PARK8*	Kinase (Endo-lysosomal trafficking)	+	+	+ PD patients	[235,236,237]
*ATP13A2* AR	*PARK9*	Lysosomal ATPase	+	+	+ PD patients	[238,239]
*FBXO7* AR	*PARK15*	Adaptor protein in E3 ubiquitin ligase subunit (Mitophagy)	+	-	+ PD patients	[225,240]
*VPS35* AD	*PARK17*	Retromer complex subunit (Vesicular trafficking)	+	+	+ PD patients	[219,240,241,242]
*GBA1*		Lysosomal hydrolase glucocerebrosidase	+	+	+ PD patients	[243,244,245,246]
*TMEM175*		Lysosomal K^+^ channel transmembrane protein	+	+	+ Rat primary neurons	[222]
*SREBF1*		Transcriptional factor (Lysosomal lipid regulation)	+	+	NA	[223,247]

Listed are PD associated genes, whose mutations cause defects in mitochondria, the endo-lysosomal system and may also cause α-synuclein (α-syn) pathology. The mutations in SNCA causes the α-syn aggregation and also leads to mitochondrial damage and inhibits autophagy processes. The parkin, PINK1, DJ-1, and FBXO7 are renowned players of mitophagy which has an effect on α-syn clearance and also impacts autophagy-endo-lysosomal pathway dysfunction. The GBA1, VPS35, ATP13A2, SREBF1, and TMEM175 are PD risk genes with functions in the endo-lysosomal system and the dysfunction of which are shown to influence mitochondrial function and Lewy body pathology. Abbreviations: NA: not available.

## Abbreviations

PD	Parkinson’s disease
APAF1	apoptotic peptidase-activating factor 1
α-syn	alpha-synuclein
Ca^2+^	calcium
CASP	caspase
DA	dopaminergic
ER	endoplasmic reticulum
ETC	electron transport chain
GD	Gaucher’s disease
H_2_O_2_	hydrogen peroxide
IM	mitochondrial inner membrane
IMS	mitochondrial intermembrane space
MCU	mitochondrial Ca^2+^ uniporter complex
Δψm	mitochondrial membrane potential
mPTP	mitochondrial permeability transition pore
MOMP	mitochondrial outer membrane permeabilization
•OH	hydroxyl radical
OM	mitochondrial outer membrane
OXPHOS	oxidative phosphorylation process
UPR^mt^	mitochondrial unfolded protein response
ROS	reactive oxygen species
O_2_^•-^	superoxide anions radical
VDAC	voltage-dependent anion channel
